# Going out with a bang: CESA7 and microtubules are required for patterning of secondary cell walls in explosive fruit

**DOI:** 10.1093/plcell/koag071

**Published:** 2026-03-17

**Authors:** Nataliia Konstantinova

**Affiliations:** Assistant Features Editor, The Plant Cell, American Society of Plant Biologists; Center for Plant Systems Biology, VIB, Gent B-9052, Belgium; Department of Plant Biotechnology and Bioinformatics, Ghent University, Gent B-9052, Belgium

Explosive seed dispersal is one of the most visually striking methods to spread seeds. Hairy bittercress (*C. hirsuta*) is a species that employs this technique, launching seeds at speeds of 10 m/s (Hofhuis et al. 2016). The seeds are ejected by ultra-fast coiling of the fruit valves, and this release mechanism depends on the formation of secondary cell walls (SCW), composed of xylan, lignin and cellulose, in endocarp b (end*b*) cells of the fruit valves.

In recent work, Eng and colleagues (Eng et al 2026) have characterized the molecular components involved in synthesis and patterning of SCW in end*b* cells of *C. hirsuta*. The authors demonstrate that CELLULOSE SYNTHASE 7 (CESA7) is required for cellulose synthesis and, together with cortical microtubules, are essential for the correct patterning and assembly of SCW polymers that drive explosive seed dispersal (Fig. 1 a).

**Figure 1:**
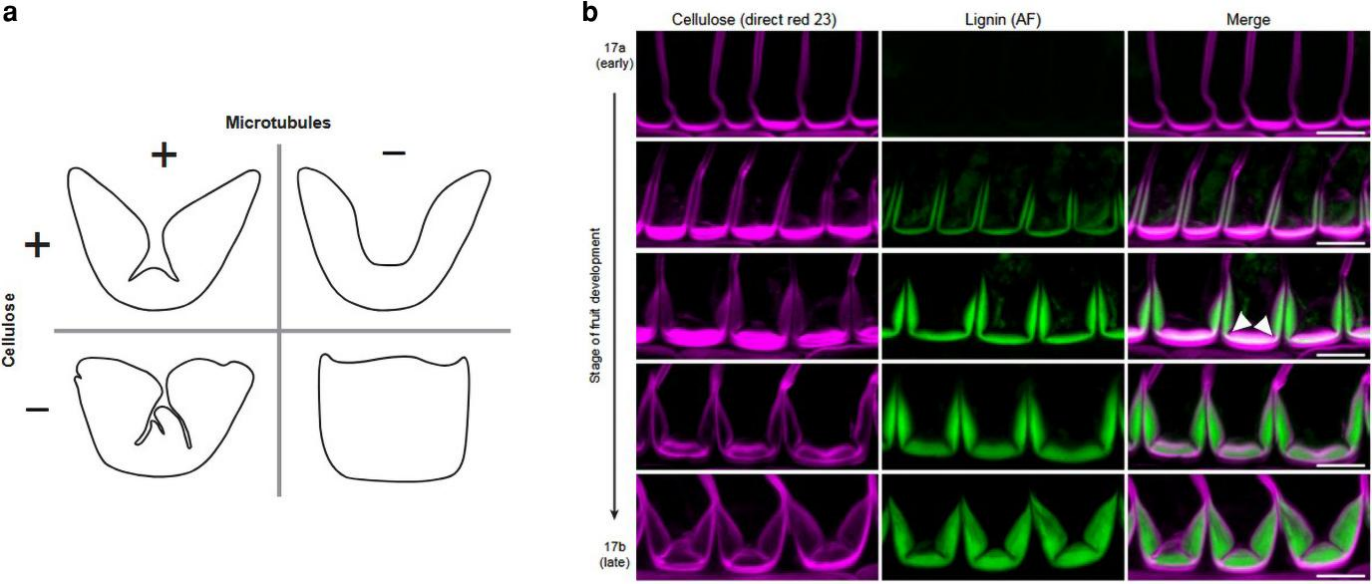
a) Visual summary showing *C.hirsuta endb* SCW deposition when either removing cellulose (*cesa7*) or depolymerizing microtubules or applying both. b) Confocal laser scanning micrographs of end*b* SCW showing deposition of cellulose (magenta) and lignin (green) across fruit development. Arrows show the hinges. Adapted from Eng et al. (2026), Figure 1B and 8B.

Performing immunofluorescence labeling targeting xyloglucan, pectin, cellulose, and lignin, the authors show that SCW synthesis happens in a coordinated manner in *C. hirsuta*. The SCW forms only on the adaxial side of end*b* cells and is progressively established through increased lignin deposition, cellulose thickening, and the formation of 2 cellulose-depleted hinges on either side (Fig. 1 b). To understand what genes control cellulose biosynthesis in these SCWs, the authors screened readily available *Arabidopsis thaliana cesa* mutants and identified *CESA7* as a causative factor. The SCW of *A. thaliana cesa7* mutants had no cellulose, and complementing this mutant with a *C. hirsuta* CESA7 construct restored cellulose biosynthesis.

The authors next generated a *C. hirsuta cesa7* mutant using CRISPR/Cas9 technology and observed the mutant to be dwarfed, with a reduction of explosive coiling in the fruit valves. Similar to the Arabidopsis *cesa7* mutant, the *C. hirsuta* mutant had collapsed xylem vessels, with irregularly shaped metaxylem pits, and very little cellulose in SCW of end*b* cells. Interestingly, the authors saw that the spatial distribution of lignin and xylan was not affected in the *cesa7* mutant, although the abundance of both was increased in the cellulose-depleted walls. Using confocal microscopy and immunolabelling, the authors show that the distinct pattern of SCW formation in end*b* cells initiates normally in *cesa7*, but the mature SCW is distorted, with incorrect assembly of xylan and thick lignin. Analyzing 3-dimensional renderings of these SCWs showed a rugged and patchy distribution of lignin in *cesa7*, while scanning electron microscopy showed loss of the layered, fibrous architecture of the SCW and failure to maintain an ordered SCW pattern.

Although the SCW end*b* patterning is lost in the *cesa7* mutant, hinge formation still allows the fruit valves to partially coil, suggesting that there are more molecular components involved. The authors next examined the role of cortical microtubules by using 2-photon excitation microscopy. They visualized the GFP-TUA6 microtubule marker in wild-type end*b* cells during SCW development, revealing that cortical microtubules are densely packed and align transversely to the long axis of the cell. They used 2 approaches to disrupt microtubule formation—oryzalin, the microtubule depolymerizing drug; and the inducible expression of a truncated tubulin kinase, PROPYZAMIDE-HYPERSENSITIVE1, which fully depolymerizes microtubules in end*b* cells. Microtubule disruption caused the hinged pattern of cellulose, lignin, and xylan in end*b* SCWs to be lost, along with the ability for fruit valves to explode.

In addition to demonstrating the importance of *CESA7* and cortical microtubules in maintaining the shape of SCW in end*b* cells of *C. hirsuta*, the authors have also shown how this explosive fruit provides a valuable system to study polar SCW patterning. It will be exciting to learn in the future, probably by combining both biomechanical analysis and computational modeling, how exactly the SCW architecture powers the process of explosive seed dispersal.

## Recent related articles in *The Plant Cell*:

- Delmer et al. (2024) reviewed the structure, biosynthesis, and function of the 5 major cell wall polymer types from birth to cell death, highlighting the historical figures that contributed to cell wall research.
- Pfaff et al (2024) developed a system to induce tracheary element transdifferentiation in isolated Arabidopsis protoplasts by transient transformation, thereby providing a new method to study secondary cell wall biosynthesis and its effect on xylem vessel formation.
- Zou et al. (2024) showed that salt induces cell wall extensin modification via hydroxyproline arabinosylation, which is necessary to modify cell wall structure and for the gravitropism response of roots.
- Zhou et al. (2024) found that in rice, ethylene induces cell wall thickening and expression of cell wall synthesis–related genes, some of which are involved in xyloglucan biosynthesis and enhanced cellulose deposition, linking ethylene hormone signaling with cell wall establishment.
